# LncRNA Rik-203 contributes to anesthesia neurotoxicity via microRNA-101a-3p and GSK-3β-mediated neural differentiation

**DOI:** 10.1038/s41598-019-42991-4

**Published:** 2019-05-02

**Authors:** Lei Zhang, Jia Yan, Qidong Liu, Zhongcong Xie, Hong Jiang

**Affiliations:** 1 Department of Anesthesiology, Shanghai Ninth People’s Hospital, Shanghai Jiao Tong University School of Medicine, Center for Specialty Strategy Research of Shanghai Jiao Tong University China Hospital Development Institute, Shanghai, P.R. China; 2 Shanghai Tenth People’s Hospital, Anesthesia and Brain Research Institute, Tongji University School of Medicine, Shanghai, P.R. China; 3 Department of Anesthesia, Critical Care and Pain Medicine, Massachusetts General Hospital and Harvard Medical School, 149 13th Street, Room, 4310 Charlestown, MA USA

**Keywords:** Neural progenitors, Developmental neurogenesis

## Abstract

The mechanism of anesthesia neurotoxicity remains largely to be determined. The effects of long noncoding RNAs (LncRNAs) on neural differentiation and the underlying mechanisms are unknown. We thus identified LncRNA Rik-203 (C130071C03Rik) and studied its role on neural differentiation and its interactions with anesthetic sevoflurane, miRNA and GSK-3β. We found that levels of Rik-203 were higher in hippocampus than other tissues and increased during neural differentiation. Sevoflurane decreased the levels of Rik-203. Rik-203 knockdown reduced mRNA levels of Sox1 and Nestin, the markers of neural progenitor cells, and decreased the count of Sox1 positive cells. RNA-RNA pull-down showed that miR-101a-3p was highly bound to Rik-203. Finally, sevoflurane, knockdown of Rik-203, and miR-101a-3p overexpression all decreased GSK-3β levels. These data suggest that Rik-203 facilitates neural differentiation by inhibiting miR-101a-3p’s ability to reduce GSK-3β levels and that LncRNAs would serve as the mechanism of the anesthesia neurotoxicity.

## Introduction

The widespread and growing use of anesthesia in children makes its safety a major health issue of interest [^1^, reviewed in^2^]. It has become a matter of even greater concern as evidence shows that multiple exposures to anesthesia and surgery may induce cognitive impairment in children^3–8^, and that anesthetics may induce neurotoxic damage and cognitive impairment in young animals^1,9–13^. These findings suggest that children who have undergone anesthesia and surgery may not develop to their full cognitive potential as they would have if they had not undergone anesthesia and surgery.

Sevoflurane, the most commonly used anesthetic in children, induces neurotoxicity and cognitive impairment in young mice^14^ and may regulate neurogenesis *in vitro*^15,16^. But, the underlying mechanism by which sevoflurane induces cognitive impairment remains largely unknown, which impedes further research into anesthesia neurotoxicity in the developing brain. Neural differentiation has been shown to contribute to cognitive impairment in young rodents^17^. Thus, in the present study, we set out to determine the effects of sevoflurane on neural differentiation and the underlying mechanisms.

Long non-coding RNAs (LncRNAs) are defined as transcripts that are longer than 200 nucleotides and are not translated into protein^18^. One of the functions of LncRNAs is to attach to microRNAs (miRNAs) as a sponge and to prevent the miRNA from binding to 3′UTR of target mRNA, thus inhibiting miRNA’s ability^19^. miRNAs are endogenous short noncoding RNAs and regulate many physiological processes by targeting mRNA 3′UTR^20–22^. LncRNAs, e.g., NBAT-1 and Pnky, may regulate cell differentiation and development [^23–25^, reviewed in^26^]. However, the role of LncRNAs on neural differentiation, the process where Embryonic Stem Cells (ESCs) mature into specialized Neural Progenitor Cells (NPCs), which is crucial for cognitive function, neural development and neurotoxicity^27^, remains largely unknown. Therefore, we used the anesthetic sevoflurane as a tool to determine the clinically relevant function of specific LncRNA and the underlying mechanism.

We identified a novel LncRNA (Rik-203: C130071C03Rik) and systematically investigated its interaction with the anesthetic sevoflurane, miRNA, the mRNA and protein of Glycogen Synthase Kinase-3β (GSK-3β). The objective of these studies was: (1) to elucidate the LncRNA-associated underlying mechanisms of anesthesia neurotoxicity; and (2) to investigate the pathway by which Rik-203 regulated neural differentiation via miR-101a-3p and GSK-3β. The hypothesis in the present studies was that the reduction of Rik-203 by sevoflurane released miR-101a-3p, which then acted on the 3′UTR of mRNA of GSK-3β, leading to reduction of mRNA of GSK-3β and consequent inhibition of neural differentiation.

## Methods

### RNA sequencing and analysis of the gene expression profiles of mRNAs, LncRNAs and miRNAs

We harvested the cells by centrifuging at 1000 × g for 2 min in the centrifuge tube. Removed the supernatant and added the RNAiso plus (Takara, China) to suspend and lysed the cells. We also harvested the mouse hippocampus tissues, and sent the cells and tissue samples protected by dry ice to the Beijing Genomics Institute (Beijing, China) for RNA-sequencing as well as the analysis of gene expression profiles of mRNA and LncRNA. In addition, we sent hippocampus tissues of mice to NovelBio (Shanghai, China) for the RNA-sequencing and analysis of gene expression profiles of the miRNA. The RNA sequencing library generation, workflow, data analysis, and enrichment analysis were performed as reported previously^28,29^. Illumina Hiseq2500/Hiseq3000 platform was used for sequencing. After trimmed using sickle.pe (pair-end) (v1.29, https://github.com/najoshi/sickle) with parameters (−q 20, −l 30), sequencing reads were mapped to genome in mouse(mm10) using Tophat (2.0.7) with the default parameters and Ensemble genome annotation (Mus_musculus.GRCm38.73.gtf)^30^. Each gene expression level (fragments per kilobase of exon per million fragments mapped) is estimated using Cufflinks (v2.0.2) software^31^. Differentially expressed genes (DEGs) were detected by Cuffdiff^32^. False discovery rate (FDR) assay is used for adjusting multiple tests. FDR < 0.05 was chosen to indicated the statistical significance.

### Establishment of inducible Rik-203 knockdown mESCs lines

We designed the specific shRNA nucleotides targeting the transcripts of LncRNA Rik-203. The primers are as follows:

shRNA-1, PF: 5′ CCGG GGTGTTGGGCCAGTTCCTTATCTCGAG ATAAGGAACTGGCCCAACACCTTTTTG-3′. PR:5′-AATTCAAAAAGGTGTTGGGCCAGTTCCTTATCTCGAGATAAGGAACTGGCCCAACACC 3′.

shRNA-2, PF: 5′-CCGGGCTTGAATTCAGGCTGCTTGACTCGAGTCAAGCAGCCTGAATTCAAGCTTTTTG-3′. PR:5′-AATTCAAAAAGCTTGAATTCAGGCTGCTTGACTCGAGTCAAGCAGCCTGAATTCAAGC-3′. Genomeditech (Shanghai, China) synthesized the oligos of shRNA-1 and shRNA-2 and cloned each of them into the pLKO-Tet-On to generate the vector of LncRNA Rik-203 shRNA1/2(Rik-203, ENSMUST00000182788.1, NR_015561.2). The full-length transcripts of LncRNA Rik-203 were cloned into the Plvx-Tight-puro vector. Specifically, the 46 C mESCs were cultured in the knockout-DMEM (Gibco, USA) medium with 15% fetal bovine serum (FBS) (Gibco, USA), 1% nonessential amino acids (NEAA) (Thermo, USA), 1% L-glutamine (Thermo, USA), 1% sodium pyruvate (Thermo, USA), 55 μM β-mercaptoethanol (Gibco, USA), and leukemia inhibitory factor (LIF) (Millipore, USA) at 37 °C, and 5% CO_2_ atmosphere. The mESCs were dissociated with 0.05% trypsin and transfected at a density of 5,000/mL with rtTA lentivirus (106 transducing units/mL) supplemented with 8 μg/mL polybrene (Qcbio Science & Technologies, China). Forty-eight hours later, cells were selected using geneticin (G418 Sulfate) (50 mg/mL, Thermo, USA). A stably transfected cell line was selected and infected with the pLKO-Tet-On-Riken shRNA lentivirus for 48 hours with 8 μg/mL polybrene before selection with 5 μg/mL puromycin (Sigma-Aldrich, USA). The medium was changed every day for 7 days until the single-cell clone could be identified under a microscope. Clones were picked up and dissociated with trypsin and plated onto feeder cell-coated 24-well plates.

### Neural differentiation of mESCs

We performed the neural differentiation of mESCs by using the methods described in previous studies^33,34^. The detail of the neural differentiation is as follows: we performed neural differentiation studies by using 46c mouse embryonic stem cells (mESCs). 46c is a Sox1-GFP reporter ESCs line that recapitulates endogenous Sox1 expression when GFP is expressed. 46C mESCs were dissociated into single cells using 0.05% trypsin (Gibco, USA) and then neutralized with DMEM (Gibco, USA) containing 10% FBS. After being counted, mESCs were washed with GMEM (Gibco, USA) and re-suspended in a Petri dish at a density of 25,000–50,000/mL using the neural differentiation medium GMEM with 8% Knockout Serum Replacement (KOSR) (Gibco, USA), 1% L-glutamine, 1% sodium pyruvate, and 0.1 mM β-mercaptoethanol. The medium was changed every 2 days.

### Sevoflurane anesthesia for treating mice and cells

C57BL/J6 mice at postnatal day 6 (P6) (Shanghai SLAC Laboratory Animal, Zhangjiang, Shanghai, P. R. China) were used in the studies. The animal protocol was approved by the Standing Committee on Animals at Shanghai Ninth People’s Hospital, Shanghai, China. All experiments were performed in accordance with relevant guidelines and regulations. According to the previous studies, the mice received the 3% sevoflurane anesthesia 2 hours daily at 6, 7, 8 day after birth to mimic the clinical several times anesthesia^14,35,36^,which is reported to induce the neurotoxicity and further cognitive function defect^2,37^. 3% is also the clinical concentration of sevoflurane for anesthesia^38^. The hippocampus tissues of mice were harvested at the end of the sevoflurane anesthesia administration. Treatment of the cells with 4.1% sevoflurane was similar to that as described in previous studies^34,39,40^. Specifically, the cells were treated with 4.1% sevoflurane for 2 hours daily at day 4, 5 and 6 after the start of neural differentiation to mimic the clinical several times anesthesia. The cells were harvested at day 7 during the neural differentiation, at which there’re many NPCs. In some experiments, the cells were transfected with GSK-3β 12 hours before the sevoflurane treatment.

### Flow cytometry studies

The cells were suspended in PBS for flow cytometry analysis by using FACS Calibur (BD Biosciences, USA) operating at 488 nm excitation with standard emission filters. Fluorescence noise baseline was referenced with the 46C mESCs. Flowjo software was used to analyze the results.

### Reverse transcription PCR and real-time quantity PCR

RNA was extracted with RNAiso Plus (TaKaRa, China). Inverse transcription of mRNA to cDNA was performed by using a cDNA Synthesis Kit (TaKaRa, China). Inverse transcription of miRNAs to cDNA was carried out with the TIANScript RT Kit (Tiangen, China). The PCR primers of miRNA were purchased (RiboBio, China). Primers for the qRT-PCR analysis of mRNA are as following sequences: Rik-203: PF: 5′-CATCACTTGGACCATGGACACTAAT-3′, RF:5′-GAATCCTATACACATGAATGCAGAA-3′; Sox1:PF:5′-GTTTTTTGTAGTTGTTACCGC-3′, RF:5′-GCATTTACAAGAAATAATAC-3′; Nestin:PF:5′-GAATGTAGAGGCAGAGAAAACT-3′, RF:5′-TCTTCAAATCTTAGTGGCTCC-3′; and GAPDH:PF: 5′-ATGACATCAAGAAGGTGGTG-3′, RF: 5′-CATACCAGGAAATGAGCTTG-3′.

### Nuclear and cytoplasm RNA extraction

We carried out the nuclear and cytoplasm extraction studies using the methods described previously^41^. Specifically, 1 × 10^8^ mESCs-derived NPCs were prepared for this assay. The cells were washed 3 times with phosphate buffered saline (PBS) and centrifuged at1,000 × g for 5 minutes. Then, lysis buffer working reagent [Tris (10 mM, pH 8.0), NaCl (140 mM), MgCl_2_ (1.5 mM) 0.5% Nonidet P-40 (NP-40)] was added to the cells and then placed into an icebox and shaken at 200 rpm on a platform for 2 hours. The samples were centrifuged at 12,000 × g for 5 min at 48 °C, and finally the nuclear and cytoplasm extract was obtained. Then, RNAiso plus (Takara) was used for RNA purification. The RNA level from cytoplasmic and nuclear was detected using quantitative RT-PCR.

### RNA pull-down assay

1 × 10^8^ mESc-derived NPCs were used for the studies. Full-length C130071C03Rik and the antisense RNA were transcribed into the cells using T7 RNA polymerase. 50 pmol of C130071C03Rik, or C130071C03Rik’s antisense RNA, was labeled using desthiobiotin and T4 RNA ligase via a PierceTM RNA 3′End Desthiobiotinylation Kit (Thermo). The RNA pull-down assay was performed according to the PierceTM Magnetic RNA-Protein Pull-Down Kit (Thermo) and parts of the experiments were performed in the core facilities in Yingbiotech (Shanghai, China). In addition, the cells were briefly lysed with Pierce IP Lysis Buffer, and incubated on ice for 5 minutes. The lysates were centrifuged at 13,000 × g for 10 minutes, and the supernatant was transferred to a new tube for further analysis. The labeled RNA was added to 50 μL of beads, and incubated for 30 minutes at room temperature with agitation. The RNA-bound beads were incubated with the lysates for 60 minutes at 4 °C. The RNA-Binding miRNAs were washed and eluted, and the binding miRNAs were detected using qRT-PCR. Primers for the qRT-PCR analysis of miRNA include the following list. Primer list of Stem-loop reverse transcription and qPCR are in the Table 1 of supplementary data.

### Luciferase assays

A pGl3-cm vector was used to construct the 3′UTR luciferase reporter. The fragment of 3′UTR was amplified from mESCs DNA by the primers in the following list. For miR-101a-3p binding sites of GSK-3β 3′ UTR region: PF: 5′-GGCGTCGACGGGGGAACAAACAGCAAACAC-3′, PR: 5′-GGCTCTAGATTTTGGCGCGTCCTGCC-3′. For miR-101a-3p binding sites of Rik-203 region: PF:5′-GGCGTCGACGAAGCTCCTATTTAGAGGAAAGGG-3′, PR:5′-GGCTCTAGAGAAACATCCTAGTTTATTTGGGGA-3′. The mutant GSK-3β 3′UTR or Rik-203 reporter vector was obtained by replacing the miR-101a-3p binding site sequences using the QuikChange Site-Directed Mutagenesis Kit (Stratagene, USA).

3T3 cells (5 × 10^4^ cells per well in 24 wells plate) were transfected with 350 ng of the 3′UTR luciferase reporter, a 5 ng Renilla vector, and 50 pmol of miR-101a-3p or miR-467a-3p mimics or control miRNA mimics (Biotend, China) using Lipofectamine 2000 (Thermo). 24 hours after the co-tansfection, the cells were harvested and the luciferase activity was analyzed using the Dual Luciferase Assay kit (Promega, USA). The luciferase activity was detected by a SpectraMax M5 microplate reader (Molecular Devices, USA).

### Western blot

Cells were lysed using SDS buffer (Beyotime, China) to obtain the protein for electrophoresis. The whole protein was transferred onto the PVDF membrane (Whatman, USA). Primary antibodies that were used in incubation include GAPDH (ab8245, Abcam), which was used for normalizing the protein levels, and GSK-3β (#12456, Cell Signaling, USA)^42–44^. Protein expression signaling was visualized through enhanced chemiluminescence (ECL) substrate (Thermo).

### Overexpression of GSK-3β by the pcDNA3.1-GSK-3β vector

The whole RNA was isolated. Inverse transcription to cDNA was performed using the cDNA Synthesis Kit (TaKaRa). GSK-3β CDS fragments were amplified and inserted into the pcDNA3.1 vector. The primer’s sequence includes the following list: PF: 5′-GGCGCTAGCATGTCGGGGCGACCGAGAA-3′ (restriction enzyme site, Nhe1), PR: 5′-GGCTCTAGATCAGGTGGAGTTGGAAGCTGAT-3′ (restriction enzyme site, Xba1). The vector was transfected into the cells by using Lipofectamine 2000 (Thermo) and the instructions for the reagent.

### Overexpression of miR-101a-3p

The pLVX-puro-miR-101-3p overexpressed vector (Biogot technology, co, Ltd, China) was transfected into the embryonic bodies derived from 46c mESCs during the neural differentiation at day 3 and day 5 using Lipofectamine 2000 (Thermo) following the instructions given to overexpress the miR-101a-3p.

### Overexpression of lncRNA Rik-203

The full length transcripts of lncRNA Rik-203 was cloned into the FUW vector the corresponding primers: 5′-GGCGGATCCTTCTCCTCTTCAACCTCCAT-3′ (BamH1 restriction enzyme), 5′-GGCGAATTCTAACATTGAAATGTATTTTTATTGA(EcoR1 restriction enzyme).

### Mutant Rik-203 overexpression vector

Mutant Rik-203 overexpression vector was constructed by replacing the miR-101a-3p seed sequence binding site of the wildtype Rik-203 overexpression vector using the QuikChange Site-Directed Mutagenesis Kit (Stratagene, USA).

### Statistics

The data were presented as mean + standard deviation (SD) with three independent experiments. The significance of statistics was determined by a Student’s t-test or one-way ANOVA. * and ^#^p < 0.05, ** and ^##^p < 0.01, *** and ^###^p < 0.001. The studies employed a two-tailed hypothesis and statistically significant p values were < 0.05. We used the Graph Pad (Software Inc., San Diego, California, USA) to evaluate all of the study data.

## Results

### Rik-203 regulated neural differentiation

**Figure 1:**
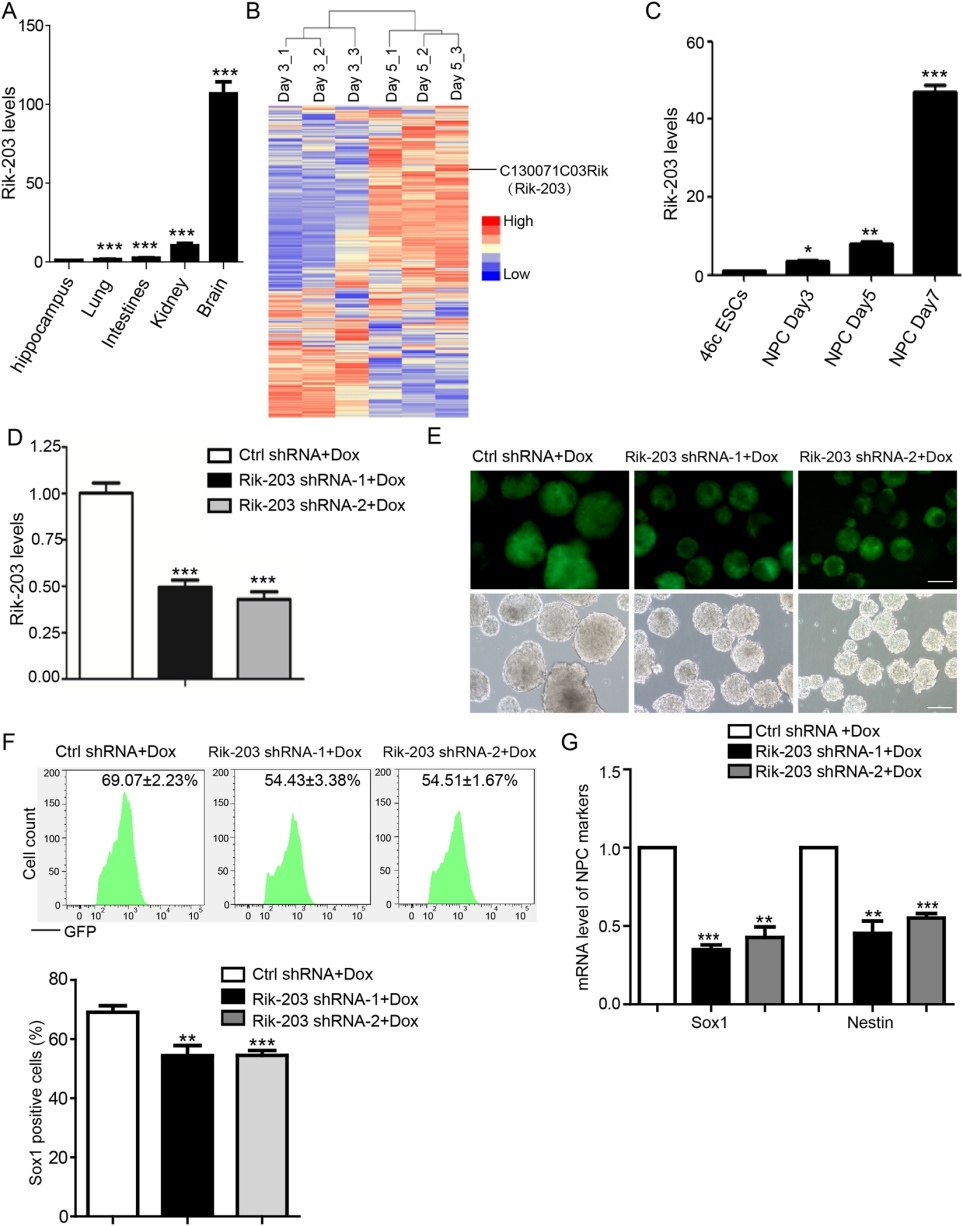
Rik-203 regulates neural differentiation. (**A**) Rik-203 levels in different tissues of mice, detected by RT-PCR. The hippocampus has the highest level of Rik-203. (**B**) Microarray studies revealed the increase of Rik-203 expression during the neural differentiation from ESCs to NPCs. (**C**) The increase of Rik-203 mRNA levels during the neural differentiation from ESCs to NPCs was confirmed by RT-PCR at day 3, 5 and 7 after the induction of neural differentiation. (**D**) knockdown of Rik-203 decreased levels of Rik-203. (**E**) Measurement of Sox1 positive cells indicated that knockdown of Rik-203 decreased the number of Sox1 positive cells. (**F**) The quantification of Sox1 positive cells using FACS showed that knockdown of Rik-203 decreased the number of Sox1 positive cells. (**G**) RT-PCR showed that the mRNA levels of Sox1 and Nestin were decreased through knockdown of Rik-203. The scale bar represents 100 um. FACS: Fluorescent-activated cell sorting. Rik-203: C130071C03Rik; ESCs: Embryonic Stem Cells; NPCs: Neural Precursor Cells; shRNA: Short hairpin RNA; GFP: Green Fluorescent Protein; Dox: Doxcycline. Ctrl: control; Sox1: SRY (sex determining region Y)-box 1. The data were presented as mean + standard deviation (SD) with three independent experiments. *p < 0.05, **p < 0.01, ***P < 0.001; by one-way ANOVA (**A**,**C**,**D**,**F**,**G**).

A recent study indicated the novel LncRNA ECONEXIN that performed the ceRNA function to promote the gliomagenesis^45^, which suggested it’s potential role of neural related regulation. Interestingly, we found that Rik-203, the ECONEXIN homologous gene in mouse, was higher expressed in the hippocampus tissues of mice than in their heart, lung, intestine and kidney tissues (Fig. 1A). We then found that there’s an increase of Rik-203 expression on day 3 and 5 after the neural differentiation from embryonic stem cells (ESCs) 46c (Fig. 1B). RT-PCR confirmed these results and demonstrated that such increases in Rik-203 levels were higher on day 7 after the induction of the neural differentiation than on days 3 and 5 (Fig. 1C). These data suggest that Rik-203 was present in higher levels in the hippocampus and that the levels increase during neural differentiation.

We established the Doxycycline (Dox) inducible RNA interference (RNAi) knockdown of Rik-203 (Fig. 1D) in the ESCs, and revealed that knockdown of Rik-203 induced by Dox begin at day 2 during the neural differentiation form the mESCs decreased the number of sex determining region Y-box 1 (Sox1) positive cells (Fig. 1E). Quantification of the Sox1 positive cells using fluorescence-activated cell sorting (FACS) showed the inhibition of neural differentiation following knockdown of Rik-203 (Fig. 1F). The knockdown of Rik-203 also decreased mRNA levels of Sox1 and Nestin, the markers of NPCs (Fig. 1G). These results suggest the role of Rik-203 in the neural differentiation process where the reduction of Rik-203 levels inhibited neural differentiation.

### The anesthetic sevoflurane decreased Rik-203 levels and the Rik-203-associated neural differentiation

**Figure 2:**
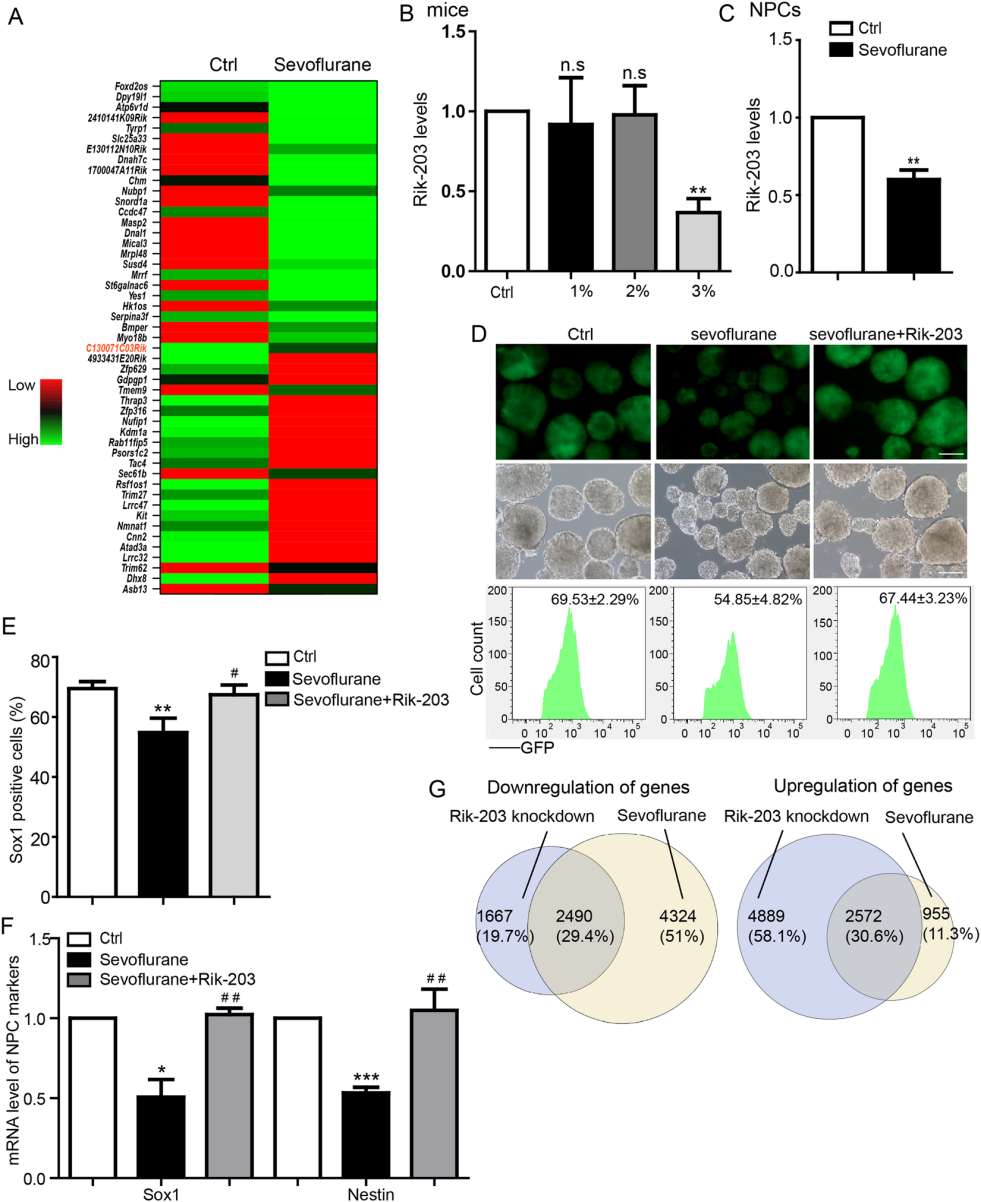
Effects of the anesthetic sevoflurane on Rik-203 levels and Rik-203-associated inhibition of neural differentiation. (**A**) RNA-seq analysis showed that 3% sevoflurane reduced the Rik-203 expression in P6 mice. (**B**) RT-PCR revealed that 3% sevoflurane decreased levels of Rik-203 in P6 mice. (**C**) RT-PCR showed that sevoflurane decreased levels of Rik-203 in NPCs derived from the ESCs. (**D**) Measurement of Sox1 showed that overexpression of Rik-203 attenuated the sevoflurane-induced reduction in Sox1 positive cells at day 7 of neural differentiation from ESCs to NPCs. (**E**) FACS showed that overexpression of Rik-203 mitigated the sevoflurane-induced reduction of Sox1 positive cells in Fig. 1D. (**F**) Overexpression of Rik-203 attenuated the sevoflurane-induced reduction in mRNA levels of Sox1 and Nestin. (**G**) RNA-seq analysis illustrated that 29.4% and 30.6% overlap of the down- and up-regulation of genes following knockdown of Rik-203 and sevoflurane treatment, respectively, in mESCs. The scale bar represents 100 um. The data were presented as mean + standard deviation (SD) with three independent experiments.* or ^#^p < 0.05, * *or ^##^p < 0.01; by *t*-test (**B**,**C**), one-way ANOVA (**E**,**F**) and hypergeometric test (**G**). Ctrl: Control; Rik-203: C130071C03Rik; NPCs: Neural Precursor Cells.

We used the anesthetic sevoflurane to further determine the clinically relevant role of Rik-203 in neural differentiation. RNA-seq analysis showed that sevoflurane decreased the levels of Rik-203 in hippocampus tissues of mice (Fig. 2A). The clinical effect of anesthesia is dose dependent. We found that sevoflurane also decreased Rik-203 mRNA levels in the hippocampus tissues of mice in a dose-dependent manner (Fig. 2B), and in NPCs (Fig. 2C).

Next, we found that sevoflurane reduced Sox1 positive cells at day 7 after the start of neural differentiation of ESCs into NPCs, and that the overexpression of Rik-203 prevented such reductions (Fig. 2D). FACS also showed that overexpression of Rik-203 mitigated the sevoflurane-induced reduction of Sox1 positive cells (Fig. 2E). Sevoflurane decreased mRNA levels of both Sox1 and Nestin, the markers of NPCs, while Rik-203 overexpression prevented sevoflurane from inducing such effects (Fig. 2F). RNA-seq analysis illustrated that 29.4% and 30.6% overlap of the down- and up-regulation of genes following knockdown of Rik-203 and sevoflurane treatment, respectively, in mESCs (Fig. 2G). Taken together, these data demonstrated the role of LncRNA Rik-203 in anesthesia neurotoxicity where the most commonly used inhalation anesthetic sevoflurane was able to regulate the levels of Rik-203 and the Rik-203-regulated neural differentiation. These data suggest that LncRNAs could be a potential novel target for research revolving the molecular mechanisms of the anesthesia neurotoxicity.

### Rik-203 regulated the function of miR-101a-3p level through a ceRNA mechanism

**Figure 3:**
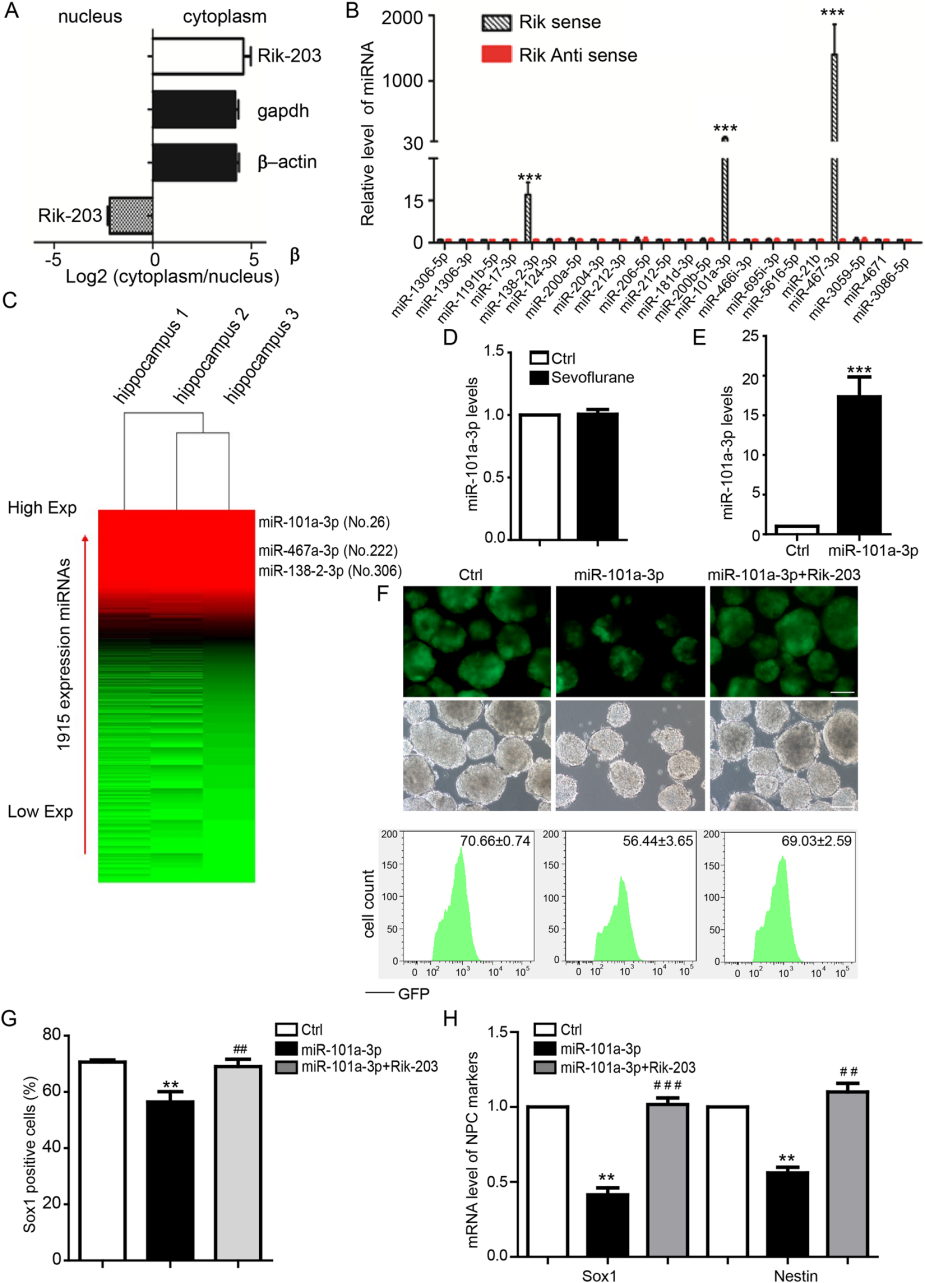
Rik-203 regulates miR-101a-3p level by sequestering miRNA through a ceRNA mechanism. (**A**) Detection of cytoplasmic and nuclear distribution of Rik-203 by fractionation. RT-PCR showed that there were higher levels of Rik-203 in the cytoplasm than in the nucleus. (**B**) RNA-RNA pull-down assay showed that miR-138-2-3p, miR-101a-3p and miR-467-3p were highly bound to Rik-203. (**C**) miR-101a was highly expressed in the hippocampus tissue. (**D**) Sevoflurane did not affect the mRNA level of miR-101a-3p in mice hippocampus tissues. (**E**) qPCR showed the ectopic expression of miR-101a effect in the NPCs. (**F**) Overexpression of Rik-203 mitigated the miR-101a-3p-induced reduction in Sox1 positive cells. (**G**) FACS analysis showed that miR-101a-3p reduced the number of Sox1 positive cells, which was mitigated by the overexpression of Rik-203. (**H**) Overexpression of Rik-203 restored the expression level of neural differentiation. The scale bar represents 100 µm. The data were presented as mean + standard deviation (SD) with three independent experiments.* or ^#^p < 0.05; ** or ^##^p < 0.01; by *t*-test (**B**,**H**,**I**) and one-way ANOVA (**E**,**F**). CeRNA: Competing endogenous RNA; NPCs: Neural Precursor Cells.

LncRNA often has different mechanisms based on its localization in cells^46^. We thus compared the levels of Rik-203 in the cytoplasm and nucleus by using RT-PCR, and found that there were higher levels of Rik-203 in the cytoplasm than in the nucleus (Fig. 3A). We found that miR-101a-3p could bind with the Rik-203 (Supplemental Fig. 1A) and then we performed a RNA pull-down assay and revealed that miR-138-2-3p, miR-101a-3p and miR-467-3p were highly bound to Rik-203 (Fig. 3B).We also performed luciferase reporter assay to detect the direct interaction of miR-101a-3p and Rik-203 and found that that overexpression of miR-101a-3p by mimics significantly repressed the luciferase activity of the reporter gene containing Rik-203 binding site,but could not influence the luciferase activity of reporter with mutant Rik-203 binding site (Supplemental Fig. 1B). These data suggest that Rik-203 within the cytoplasm may attach to miRNA. We also found that there were higher levels of miR-101a-3p in the hippocampus than those of miR-138-2-3p and miR-467a-3p. Specifically, miR-101a-3p was ranked the 26^th^ among the 1915 expressions of miRNAs in the mice hippocampus tissues (Fig. 3C). We also found that sevoflurane did not affect the levels of miR-101a-3p (Fig. 3D). These results suggest that sevoflurane likely acts on Rik-203, but not on miR-101a-3p, to decrease neural differentiation. Collectively, these findings support the competing endogenous RNA (ceRNA) hypothesis that Rik-203 may serve as a “sponge” to tie with miRNAs and prevent the binding of miRNAs to their target mRNAs. By overexpressing miR-101a-3p (Fig. 3E), we found that miR-101a-3p decreased the Sox1 positive cells whereas in contrast the overexpression of Rik-203 mitigated such decreases (Fig. 3F). FACS studies further indicated that miR-101a-3p reduced Sox1 positive cells, and that overexpression of Rik-203 mitigated such reductions (Fig. 3G). Furthermore, overexpression of miR-101a-3p reduced the mRNA levels of the NPC markers Sox1 and Nestin, which were mitigated by the overexpression of Rik-203 (Fig. 3H). These findings suggest that Rik-203 can bind to and interact with miR-101a-3p, leading to the facilitation of neural differentiation. We also found that miR-467a-3p inhibited neural differentiation (Supplemental Fig. 1C). Given the fact that miR-101a-3p has higher levels in the hippocampus than miR-467a-3p, we focused solely on determining the effects of miR-101a-3p on neural differentiation and its interaction with Rik-203.

### MiR-101a-3p targeted GSK-3β to mediate the Rik-203-associated neural differentiation

**Figure 4:**
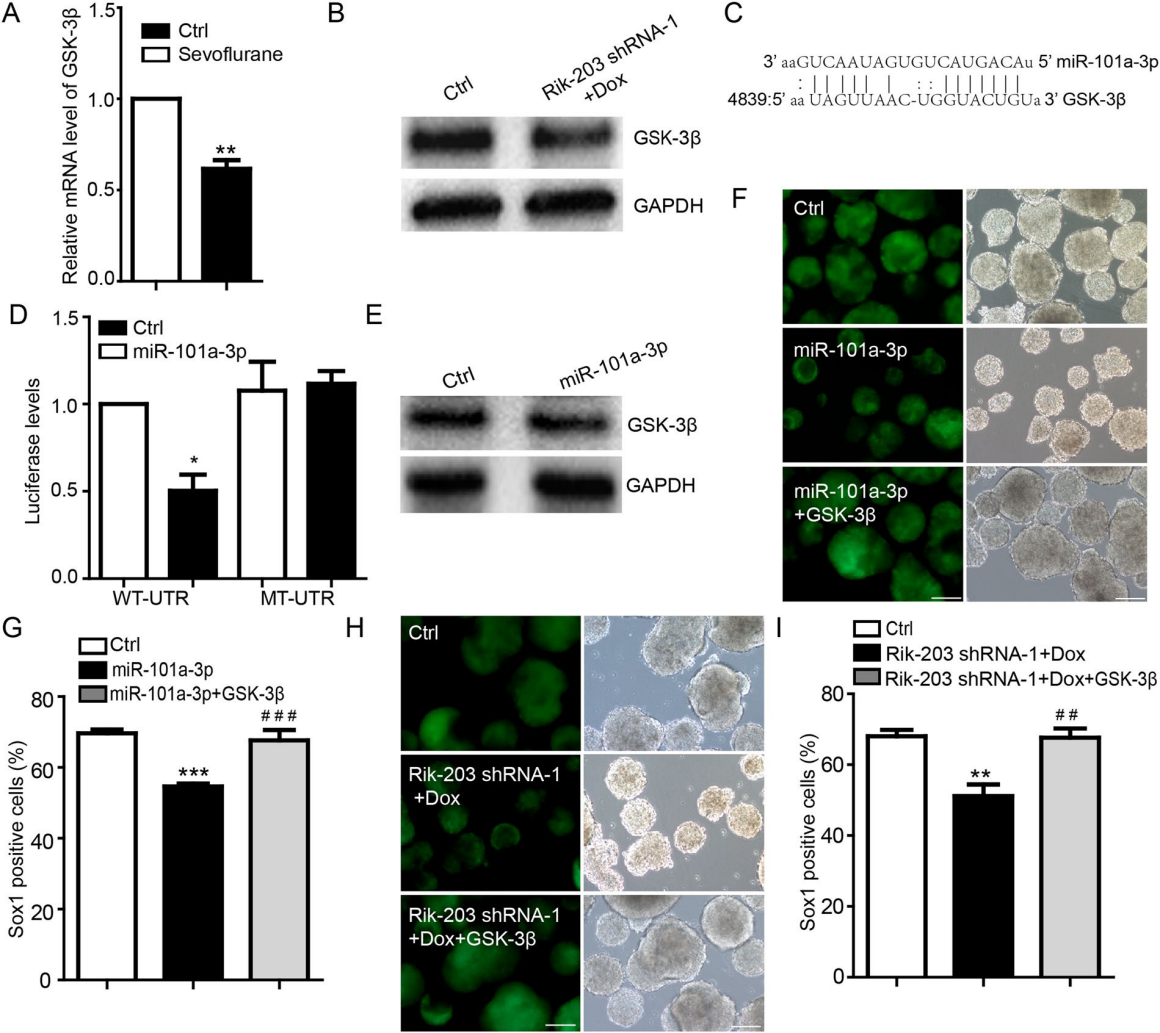
miR-101a-3p targets GSK-3β to mediate the Rik-203-associated neural differentiation. (**A**) qRT-PCR detection showed that sevoflurane decreased the expression of GSK-3β. (**B**) Knockdown of Rik-203 decreased the protein levels of GSK-3β in NPCs. (**C**) Target validation of the binding of GSK-3β 3′UTR by miR-101a-3p. (**D**) Luciferase report assay indicated that miR-101a-3p targeted wild-type GSK-3β 3′UTR but not mutant UTR (deletion of the miRNA binding seed sequence). (**E**) Overexpression of miR-101a-3p decreased the protein level of GSK-3β. (**F**) Overexpression of GSK-3β mitigated the miR-101a-3p-induced reduction in Sox1 positive cells. (**G**) FACS analysis showed that overexpression of GSK-3β mitigated the miR-101a-3p-induced decrease in the levels of Sox1 positive cells. (**H**) Overexpression of GSK-3β mitigated the knockdown of Rik-203-induced reduction in Sox1 positive cells. (**I**) FACS analysis showed that overexpression of GSK-3β mitigated the knockdown of Rik-203-induced decrease in the levels of Sox1 positive cells. The scale bar represents 100 µm. The data were presented as mean + standard deviation (SD) with three independent experiments.* or ^#^p < 0.05; * *or ^##^p < 0.01; by *t*-test (**D**,**E**) and one-way ANOVA (**H**,**J**,**L**). GSK-3β: Glycogen synthase kinase-3β; GAPDH: Glyceraldehyde-3-phosphate dehydrogenase; NPCs: Neural Precursor Cells.

GSK-3β has been shown to be regulated by the anesthetic sevoflurane^47^ and linked to miR-101a-3p^48^. Thus, we assessed the interactions of sevoflurane, Rik-203, miRNA, and GSK-3β. We employed a RNA-seq in the experiments and found that sevoflurane decreased mRNA levels of GSK-3β (Fig. 4A). We performed the doxycycline (Dox) inducible knockdown of Rik-203 at day 2 after the Dox induction and found that Gsk-3β was downregulated during the neural differentiation detected at day 4 (Fig. 4B). Using the online miRNA target predication software TargetScan^49^ and miRanda^50^, we predicted that GSK-3β might be targeted by miR-101a-3p (Fig. 4C). These data revealed the role of Rik-203 in the metabolism of GSK-3β and suggest that sevoflurane may regulate GSK-3β levels by acting on Rik-203.

Next, we examined the interaction of miRNA with sevoflurane, Rik-203 and GSK-3β. We engineered luciferase reporters that had the wild-type 3′UTRs of GSK-3β or the mutant UTRs without the miRNA seed sequence-binding site. The luciferase report assay indicated that miR-101a-3p (Fig. 4D) targeted wild-type GSK-3β 3′UTR but not mutant UTR (deletion of the miRNA binding seed sequence). We were able to show that overexpression of miR-101a-3p decreased the protein levels of GSK-3β (Fig. 4E). Overexpression of GSK-3β mitigated the miR-101a-3p-induced reduction of the mRNA levels of GSK-3β (Supplemental Fig. 2A).

We found that the overexpression of miR-101a-3p reduced the number of Sox1 positive cells (Fig. 4F,G) and the mRNA levels of Sox1 and Nestin (Supplemental Fig. 2B,C), which was also mitigated by the overexpression of GSK-3β. Additionally, We mutant the Rik-203 overexpression vector by replacing the miR-101a-3p binding site with the sequence that was the same as miR-101a-3p seed sequence. Then we found that overexpression of wild type but not mutant Rik-203 could restored the GSK-3β downregulated by miR-101a-3p. (Supplementary Fig. 2D).Overexpression of GSK-3β mitigated the knockdown of Rik-203-induced decrease of mRNA levels of GSK-3β (Supplemental Fig. 2E) and the Sox1 positive cells (Fig. 4H,I, Supplementary Fig. 2F), and reduced mRNA levels of Sox1 and Nestin (Supplementary Fig. 2G).

## Discussion

Sevoflurane has extensive regulation effect to tissues by different physiological processes. Previous studies showed that sevoflurane impairs insulin secretion, to induce insulin resistance^51^. Administration of sevoflurane before cardiopulmonary bypass induced cardioprotection in patients undergoing coronary artery bypass graft surgery^52^. However, sevoflurane also was reported to inhibit cardiac function in pulmonary fibrosis mice^53^. These studies suggested the toxicity of sevoflurane is systemically and complex. Previous study indicated that infants received multiple but not single anesthesiology have higher increased risk of further cognitive impair^54,55^. In the current studies, we mimic the clinical interval multiple anesthesiology operation to treated the mouse with sevoflurane (3%) plus 60% oxygen (balanced with nitrogen) 2 h daily for 3 consecutive days as performed in previous studies^14,47^. We showed for the first time that the anesthetic sevoflurane decreased levels of LncRNA Rik-203 in the hippocampus tissues of the mice. Such reductions resulted in the inhibition of neural differentiation via the cascade action of miRNA (miR-101a-3p) and GSK-3β. These data showed the clinical and physiological relevance effects of Rik-203 and suggest that LncRNA Rik-203 would serve as the underling mechanism for anesthesia neurotoxicity.

The mechanics insight of the current studies was that Rik-203, a hippocampus rich LncRNA located in the cytoplasm, interacted with miR-101a-3p and served as a “sponge” to compete with downstream target mRNAs for the binding with miR-101a-3p. The reduction of Rik-203, by knockdown or by sevoflurane, released miR-101a-3p, which then acted on the 3′UTR of mRNA of GSK-3β, leading to reduction of mRNA of GSK-3β and consequent inhibition of neural differentiation (Supplemental Fig. 3A,B).

LncRNA C130071C03Rik has 5 transcripts (splice variants). A recent study identified a novel LncRNA Rik-201 and demonstrated its functional role in gliomagenesis^45^. The findings from the current study showed, for the first time, that Rik-203 contributed to neural differentiation through acting on miRNA and GSK-3β. Although other studies have reported that miR-101a-3p regulates GSK-3β activity in the glioblastoma^48^, the role of miR-101a-3p in regulating neural differentiation was first reported in this study.

LncRNAs are known to function as epigenetic modulators to orchestrate epigenetic processes^56^. Although LncRNAs have been found to play crucial roles in developmental and neurodegenerative diseases^57^, their function in anesthesia-induced influence is not very clear. Some LncRNAs expression has been reported to be associated with the sevoflurane. LncRNA Gadd45a upregulation is associated with sevoflurane-induced neurotoxicity in rat neural stem cells^58^. LINC00652 reduce the protective effect of sevoflurane on myocardial ischemia-reperfusion injury in mice^59^. These studies suggested the complex regulation of sevoflurane to lncRNAs and indicated the potential different signaling pathway of sevoflurne /LncRNAs axis. Our studies showed that sevoflurane decreased the levels of Rik-203, which is mainly located in the cytoplasm, and inhibited neural differentiation via its downstream effects on miR-101a-3p and GSK-3β.

Lu *et al*. also reported that sevoflurane was able to increase the level of LncRNA Gadd45a in the rat hippocampus neural stem cells^58^. However, the studies to determine the downstream effects and the underlying mechanisms were not performed. Our studies specifically showed that sevoflurane decreased Rik-203 levels, leading to miRNA- and GSK-3β-regulated inhibition of neural differentiation.

Several studies found that sevoflurane caused neurotoxicity by directly regulating the expression of miRNAs^16,60,61^. In the present study, we showed that sevoflurane might still regulate the function of miRNA without directly affecting the levels of miRNA (Fig. 3). Rather, sevoflurane decreased the levels of Rik-203, which led to the release of the miR-101a-3p from Rik-203. The released miR-101a-3p then decreased the levels of GSK-3β, leading to the inhibition of the neural differentiation. Additionally, miR-9 is widely studied in the neural related physiological progress and reported to be necessary for neural differentiation^62–64^. Here we found that full length of lncRNA C13007AC03 Rik has intersection with miR-9, but the variant Rik-203 has no intersection with miR-9.

Specifically, the reduction of Rik-203, by knockdown or by sevoflurane, inhibited the “sponge” function of Rik-203, which then released miR-101a-3p to reduce GSK-3β levels and led to the inhibition of neural differentiation (Supplemental Fig. 3). Moreover, both miR-101a-3p^65^ and sevoflurane^40,47^ have been reported to increase interleukin-6 (IL-6) levels. These findings suggest that sevoflurane may induce other effects, e.g., neuroinflammation, via regulating miRNA, through LncRNAs, e.g., like Rik-203. Future studies to test this hypothesis are warranted.

There are several limitations in the studies. First, we did not perform *in vivo* relevance studies on the *in vitro* findings of the cascade of “sevoflurane, Rik-203, miR-101a-3p, GSK3β and neural differentiation”. However, the data from the present studies demonstrated that sevoflurane could inhibit neural differentiation via LncRNAs and miRNA. Second, Rik-203 bound to miR-138-2-3p, miR-101a-3p and miR-467a-3p. However, we did not perform downstream studies of miR-138-2-3p and miR-467a-3p.

In conclusion, we identified the functional role of LncRNA Rik-203 in facilitating neural differentiation and elucidated the underlying miRNA-GSK-3β-associated molecular mechanisms, which could promote further studies of the role of LncRNA on neural differentiation.

## Supplementary information

Supplemental data
