# Supplementary material for: LncRNA Rik-203 contributes to anesthesia neurotoxicity via microRNA-101a-3p and GSK-3β-mediated neural differentiation
Source: Sci Rep. 2019 May 2;9:6822. doi: 10.1038/s41598-019-42991-4 (PMC6497879; doi:10.1038/s41598-019-42991-4)
Supplement: Supplementary file 1 — Supplemental data [file 41598_2019_42991_MOESM1_ESM.pdf]

**Supplemental data**

**LncRNA Rik-203 contributes to anesthesia neurotoxicity viamicroRNA-101a-3p and GSK-3β-mediated neural differentiation**

Lei Zhang, Jia Yan, Qidong Liu, ZhongcongXie and Hong Jiang

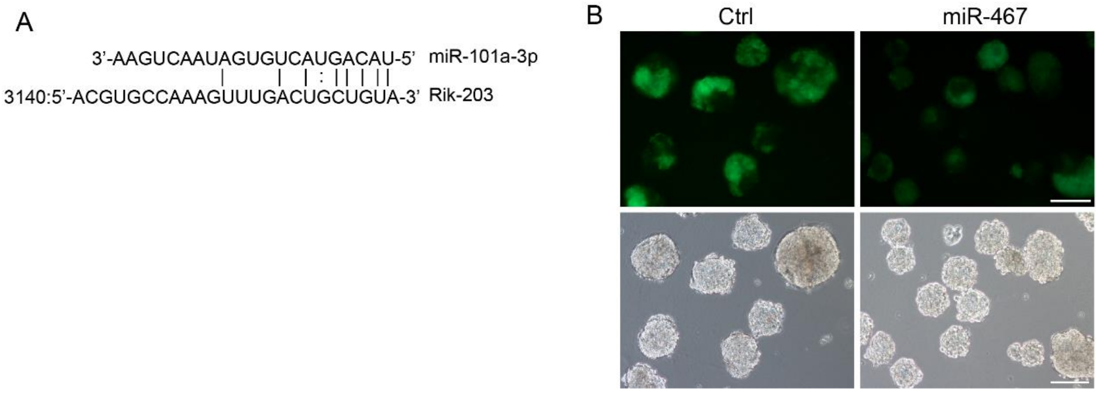


**Supplementary Fig. 1. miR-467a-3p inhibited neural differentiation**.

**A.** Target validation of the miR-101a-3p binding to Rik-203.

**B**.Overexpression of miR-467a-3p inhibited neural differentiation. Scale bar represents 100µm.

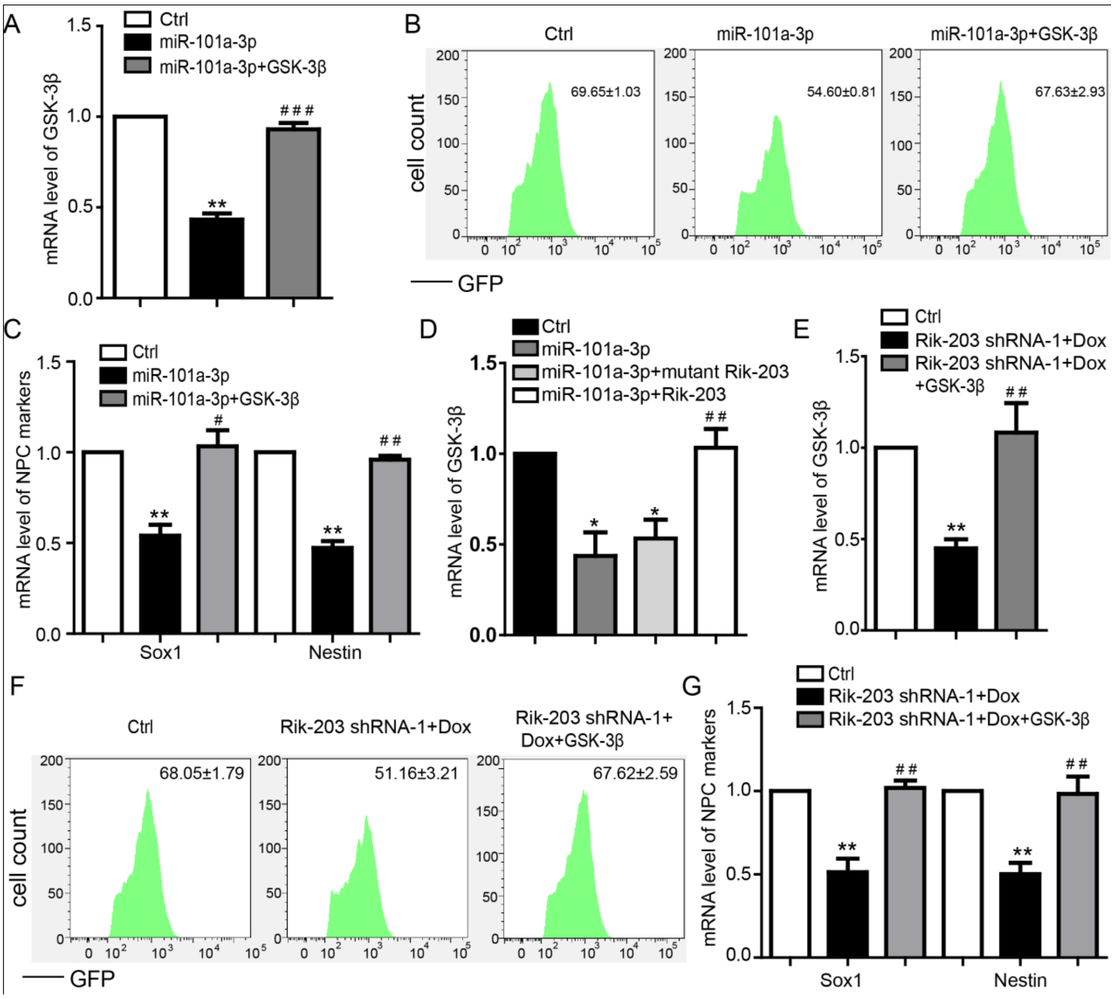


**Supplementary Fig. 2**

**Supplementary Fig 2. The Rik-203/miR-101a-3p/ GSK-3β signaling axis regulates neural differentiation.A**. Expression of GSK-3β mRNA level was decreased by overexpression of miR-101a-3p and restored by further overexpression of GSK-3β. **B**. The quantification of Sox1 positive cells detected by using FACS.**C**. miR-101a-3p decreased the mRNA levels of both Sox1 and Nestin. Overexpression of GSK-3β mitigated such decreases. **D**. Overexpression of wild type but not mutant Rik-203 could restored the GSK-3β downregulated by miR-101a-3p.**E.**GSK-3β mRNA level was decreased by knockdown of Rik-203 and mitigated by ectopic expression of GSK-3β.**F.**The quantification of Sox1 positive cells using FACS.**G**. Knockdown of Rik-203 decreased the Sox1 and Nestin mRNA levels which was mitigated by overexpression of GSK-3β. The data were presented as mean ± standard deviation (SD) with three independent experiments.* or $^{\#}$P <0.05,** or $^{\#\#}$P < 0.01;

by one-way ANOVA test (A-F).Rik-203:C130071C03Riken; ESCs:Embryonic Stem Cells; NPCs:Neural Precursor Cells; shRNA: Short hairpin RNA;GFP: Green Fluorescent Protein;GSK-3β : Glycogen synthase kinase-3β;GAPDH:Glyceraldehyde-3-phosphate dehydrogenase.

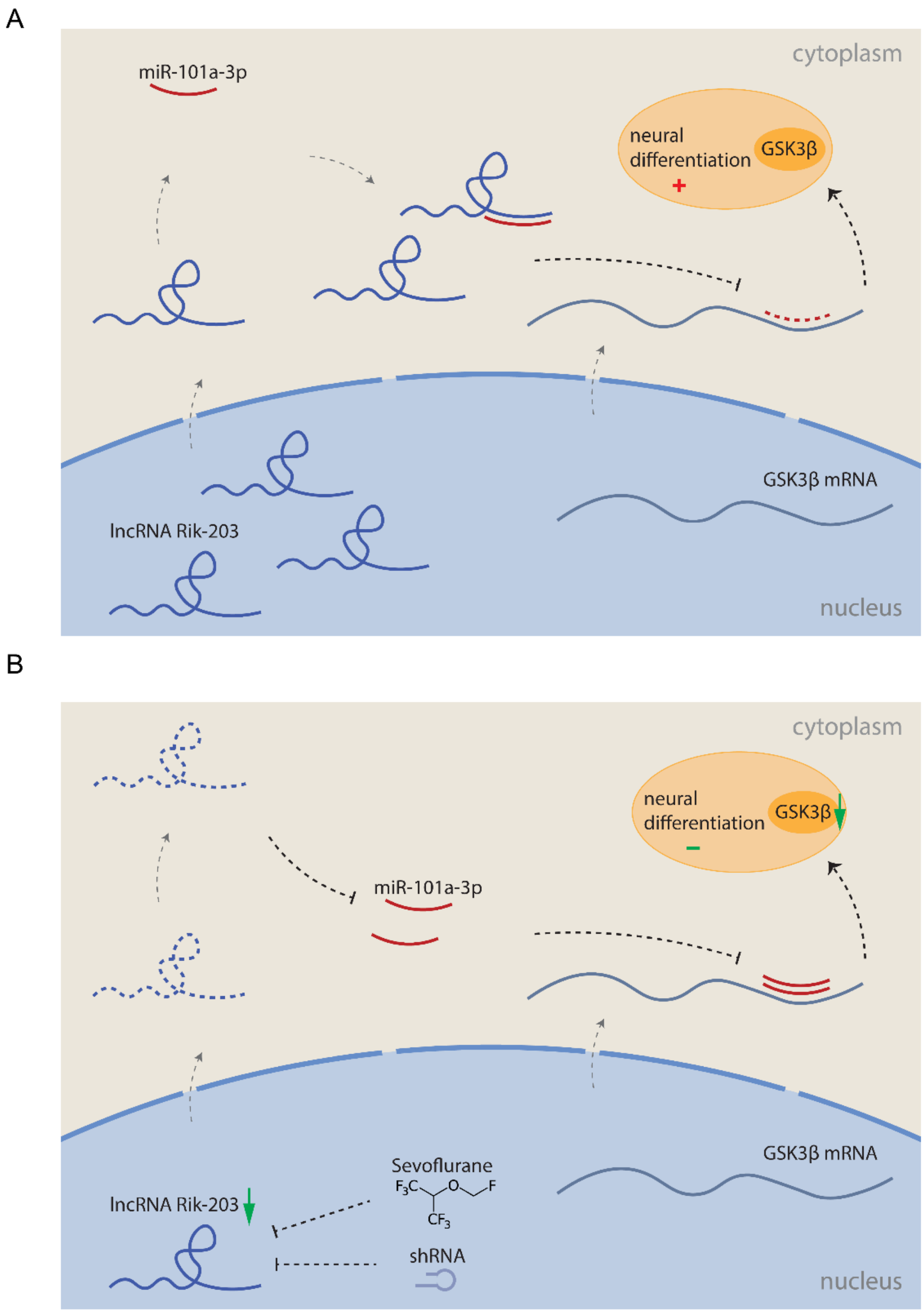


**Supplementary Fig. 3 Hypothesized pathway.**

Rik-203 in cytoplasm bind to miR-101a-3p, which inhibit their downstream function, leading to facilitation of neural differentiation. **B**. Reduction of Rik-203 following knockdown of Rik-203 or sevoflurane treatment enhances the action of miR-101a-3p on reducing GSK-3β levels, leading to inhibition of neural differentiation.

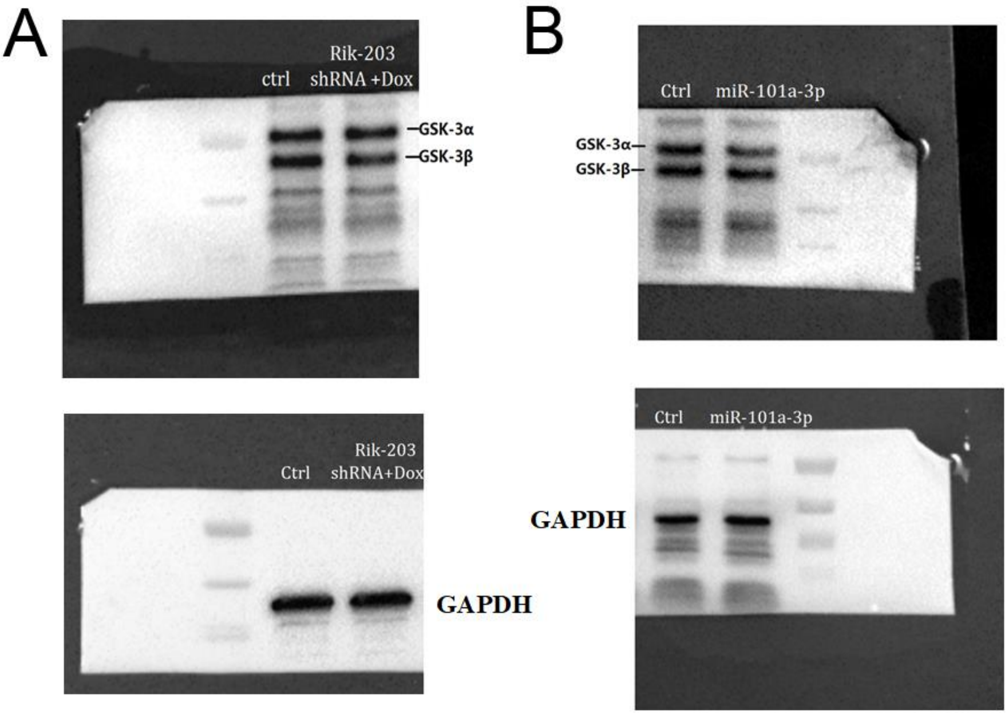


**Supplementary Fig. 4**

**The uncropped western blot pictures of figure 4B and 4E**

A.figure 4B GSK-3βand figure 4B GAPDH.B. figure 4E GSK-3β and figure 4E GAPDH

**Table1　Primers for RNA-pull down RT-PCR assay**

| miRNA | RT-primer | Forward primer | Reverse primer |
|---|---|---|---|

| | | | |
|---|---|---|---|
| mmu-miR-1306-5p | 5'GTCGTATCCAGTGCGTGTCGTGGAGTCGGCAATTGCACTGGATACGACGGACGTT3' | 5'AGTGCGTGTCGTGGAGTCG 3' | 5'AGACGCACCACCTCCCCT3' |
| mmu-miR-1306-3p | 5'GTCGTATCCAGTGCGTGTCGTGGAGTCGGCAATTGCACTGGATACGACCATCACC3' | 5'AGTGCGTGTCGTGGAGTCG 3' | 5'AGACGACGTTGGCTCTGGTG 3' |
| mmu-miR-1191b-5p | 5'GTCGTATCCAGTGCGTGTCGTGGAGTCGGCAATTGCACTGGATACGACAGGTTCT 3' | 5'AGTGCGTGTCGTGGAGTCG 3' | 5'GCGGTCAGGCTACAGAGCGA3' |
| mmu-miR-17-3p | 5'GTCGTATCCAGTGCGTGTCGTGGAGTCGGCAATTGCACTGGATACGACCTACAAG3' | 5'AGTGCGTGTCGTGGAGTCG 3' | 5'ACGACTGCAGTGAGGGCACT3' |
| mmu-miR-124-3p | 5'GTCGTATCCAGTGCGTGTCGTGGAGTCGGCAATTGCACTGGATACGACGGCATTC3' | 5'AGTGCGTGTCGTGGAGTCG 3' | 5'GAGACGTAAGGCACGCGGT3' |
| mmu-miR-138-2-3p | 5'GTCGTATCCAGTGCGTGTCGTGGAGTCGGCAATTGCACTGGATACGACACCCTGG3' | 5'AGTGCGTGTCGTGGAGTCG 3' | 5'CGGCGGGCTATTTCACGA3' |
| mmu-miR-200a-5p | 5'GTCGTATCCAGTGCGTGTCGTGGAGTCGGCAATTGCACTGGATACGACTCCAGCA3' | 5'AGTGCGTGTCGTGGAGTCG 3' | 5'AGACGCATCTTACCGGACAGTG3' |
| mmu-miR-204-3p | 5'GTCGTATCCAGTGCGTGTCGTGGAGTCGGCAATTGCACTGGATACGACACGTCCC 3' | 5'AGTGCGTGTCGTGGAGTCG 3' | 5'AGACGGCTGGGAAGGCAA3' |
| mmu-miR-181d-3p | 5' GTCGTATCCAGTGCGTGTCGTGGAGTCGGCAATTGCACTGGATACGACTGACATT 3' | 5'AGTGCGTGTCGTGGAGTCG 3' | 5'AACCCACCGGGGGATGA3' |
| mmu-miR-212-3p | 5' GTCGTATCCAGTGCGTGTCGTGGAGTCGGCAATTGCACTGGATACGACTGGCCGT 3' | 5'AGTGCGTGTCGTGGAGTCG 3' | 5' GGCGGTAACAGTCTCCAGTCA 3' |
| mmu-miR-212-5p | 5' GTCGTATCCAGTGCGTGTCGTGGAGTCGGCAATTGCACTGGATACGACAGTAAGC 3' | 5'AGTGCGTGTCGTGGAGTCG 3' | 5'CGGCGGACCTTGGCTCTA 3' |
| mmu-miR-206-5p | 5' GTCGTATCCAGTGCGTGTCGTGGAGTCGGCAATTGCACTGGATACGACTATGAGG 3' | 5'AGTGCGTGTCGTGGAGTCG 3' | 5' CGGCGGACATGCTTCTTTATAT 3' |
| mmu-miR-200b-5p | 5' GTCGTATCCAGTGCGTGTCGTGGAGTCGGCAATTGCACTGGATACGACTCCAATG 3' | 5'AGTGCGTGTCGTGGAGTCG 3' | 5' CGCATCTTACTGGGCAGCA 3' |
| mmu-miR-101a-3p | 5'GTCGTATCCAGTGCGTGTCGTGGAGTCGGCAATTGCACTGGATACGACTTCAGTT 3' | 5'AGTGCGTGTCGTGGAGTCG 3' | 5'GCGGCGGTACAGTACTGTGATA 3' |
| mmu-miR-466i-3p | 5'GTCGTATCCAGTGCGTGTCGTGGAGTCGGCAATTGCACTGGATACGACT | 5'AGTGCGTGTCGTGGAGTCG | 5'CGGCGGATACAC |

| | | | |
|---|---|---|---|
| | AGTGTG 3' | 3' | ACACACATA 3' |
| mmu-miR-6951-3p | 5'GTCGTATCCAGTGCGTGTCGTGGAGTCGGCAATTGCACTGGATACGACCTGTATT 3' | 5'AGTGCGTGTCGTGGAGTCG 3' | 5'CGGCGGCTTTTTTCTTCACA 3' |
| mmu-miR-5616-5p | 5'GTCGTATCCAGTGCGTGTCGTGGAGTCGGCAATTGCACTGGATACGACTCAACTT3' | 5'AGTGCGTGTCGTGGAGTCG 3' | 5'CGGCGGTTTCCTCTCATCAC3' |
| mmu-miR-21b | 5'GTCGTATCCAGTGCGTGTCGTGGAGTCGGCAATTGCACTGGATACGACGGAAATA 3' | 5'AGTGCGTGTCGTGGAGTCG 3' | 5'CGGCGGTAGTTTATCAGACTGA 3' |
| mmu-miR-467a-3p | 5'GTCGTATCCAGTGCGTGTCGTGGAGTCGGCAATTGCACTGGATACGACTGTAGGT 3' | 5'AGTGCGTGTCGTGGAGTCG 3' | 5'CGGCGGCATATACATACACACA 3' |
| mmu-miR-3059-5p | 5'GTCGTATCCAGTGCGTGTCGTGGAGTCGGCAATTGCACTGGATACGACACCCTAT 3' | 5'AGTGCGTGTCGTGGAGTCG 3' | 5'CAGTTTCCTCTCTGCCCCA 3' |
| mmu-miR-467f | 5'GTCGTATCCAGTGCGTGTCGTGGAGTCGGCAATTGCACTGGATACGACTGTAGGT 3' | 5'AGTGCGTGTCGTGGAGTCG 3' | 5'GCAGCGCAGATATACACACACAC 3' |
| mmu-miR-3086-5p | 5'GTCGTATCCAGTGCGTGTCGTGGAGTCGGCAATTGCACTGGATACGACTCCAATG 3' | 5'AGTGCGTGTCGTGGAGTCG 3' | 5'GCGGCGGTAGATTGTAGGC 3' |
